# Simultaneous detection of eight avian influenza A virus subtypes by multiplex reverse transcription-PCR using a GeXP analyser

**DOI:** 10.1038/s41598-018-24620-8

**Published:** 2018-04-18

**Authors:** Meng Li, Zhixun Xie, Zhiqin Xie, Jiabo Liu, Liji Xie, Xianwen Deng, Sisi Luo, Qing Fan, Li Huang, Jiaoling Huang, Yanfang Zhang, Tingting Zeng, Sheng Wang

**Affiliations:** Guangxi Key Laboratory of Veterinary Biotechnology, Guangxi Veterinary Research Institute, 51 You Ai North Road, Nanning, Guangxi 530001 China

## Abstract

Recent studies have demonstrated that at least eight subtypes of avian influenza virus (AIV) can infect humans, including H1, H2, H3, H5, H6, H7, H9 and H10. A GeXP analyser-based multiplex reverse transcription (RT)-PCR (GeXP-multiplex RT-PCR) assay was developed in our recent studies to simultaneously detect these eight AIV subtypes using the haemagglutinin (HA) gene. The assay consists of chimeric primer-based PCR amplification with fluorescent labelling and capillary electrophoresis separation. RNA was extracted from chick embryo allantoic fluid or liquid cultures of viral isolates. In addition, RNA synthesised via *in vitro* transcription was used to determine the specificity and sensitivity of the assay. After selecting the primer pairs, their concentrations and GeXP-multiplex RT-PCR conditions were optimised. The established GeXP-multiplex RT-PCR assay can detect as few as 100 copies of premixed RNA templates. In the present study, 120 clinical specimens collected from domestic poultry at live bird markets and from wild birds were used to evaluate the performance of the assay. The GeXP-multiplex RT-PCR assay specificity was the same as that of conventional RT-PCR. Thus, the GeXP-multiplex RT-PCR assay is a rapid and relatively high-throughput method for detecting and identifying eight AIV subtypes that may infect humans.

## Introduction

Influenza A viruses are important human and animal pathogens affecting human health, causing severe animal diseases and death. To date, 16 HA (haemagglutinin) types and nine NA (neuraminidase) influenza A viral types have been identified based on the combination of these two major antigens in avian populations. The 17th and 18th HA types and 10th and 11th NA types were recently discovered in bats in South America^1–4^. Avian influenza (AI) is an acute infectious disease caused by influenza A viruses or avian influenza viruses (AIVs) in domestic poultry and wild birds. Some AIV subtypes can break the species barrier and infect humans^5,6]^. Studies have shown that AIV subtypes H1, H2, H3, H5, H7, H9 and H10 can directly infect humans and could be potentially lethal pathogens that cause human influenza pandemics. Research has shown that most human influenza viruses originate from AIVs. Prior to 2013, human AIV infection cases included infections by H5N1, H5N2, H7N2, H7N3, H7N7, H9N2 and H10N7^7–10^. Since 2013, four additional AIV subtypes, H7N9, H6N1, H10N8 and H5N6, have been detected in humans, and new cases of these subtypes continue to appear in China. For example, H7N9 AIV can infect humans and poultry but has low pathogenicity in chickens. In 2017, H7N9 AIV mutated into a strain that is highly pathogenic against chickens and caused hundreds of cases of human infections in China^11–14^. To date, at least eight AIV subtypes, including H1, H2, H3, H5, H6, H7, H9 and H10, have been reported to infect humans^15^.

Effective laboratory techniques are necessary to detect and identify AIV subtypes during outbreaks. Molecular biological diagnostic methods based on PCR technology, such as conventional PCR, reverse transcription PCR (RT-PCR), real-time RT-PCR (RRT-PCR) and quantitative RRT-PCR have been widely used for AIV detection and genotyping^16–19^. However, a method is needed that uses only one PCR reaction to simultaneously detect and differentiate all HA subtypes of AIV strains that can infect humans. In general, RRT-PCR and multiplex RT-PCR can detect multiple AIV pathogenic subtypes^20–22^ but no more than four at once. The GeXP analyser is an instrument that can detect the expression of up to 35 genes simultaneously using a multiplex gene expression profiling analysis platform (Beckman Coulter, Brea, CA, USA). Several human and animal pathogens, including those causing hand, foot and mouth disease, 16 human respiratory viral types or subtypes, and 11 human papilloma viruses have been successfully and rapidly detected and identified using the GeXP analyser^23–25^. Moreover, our laboratory also developed several procedures to simultaneously detect nine avian respiratory pathogens, eight swine reproductive and respiratory viruses, eleven duck viruses, six immunosuppressive chicken viruses and four different avian influenza A H5 NA viral types^26–30^. This report describes our recently developed multiplex RT-PCR assay using a GeXP analyser (GeXP-multiplex RT-PCR) to simultaneously detect eight AIV subtypes that can infect humans.

## Results

### GeXP-multiplex RT-PCR specificity testing

**Figure 1:**
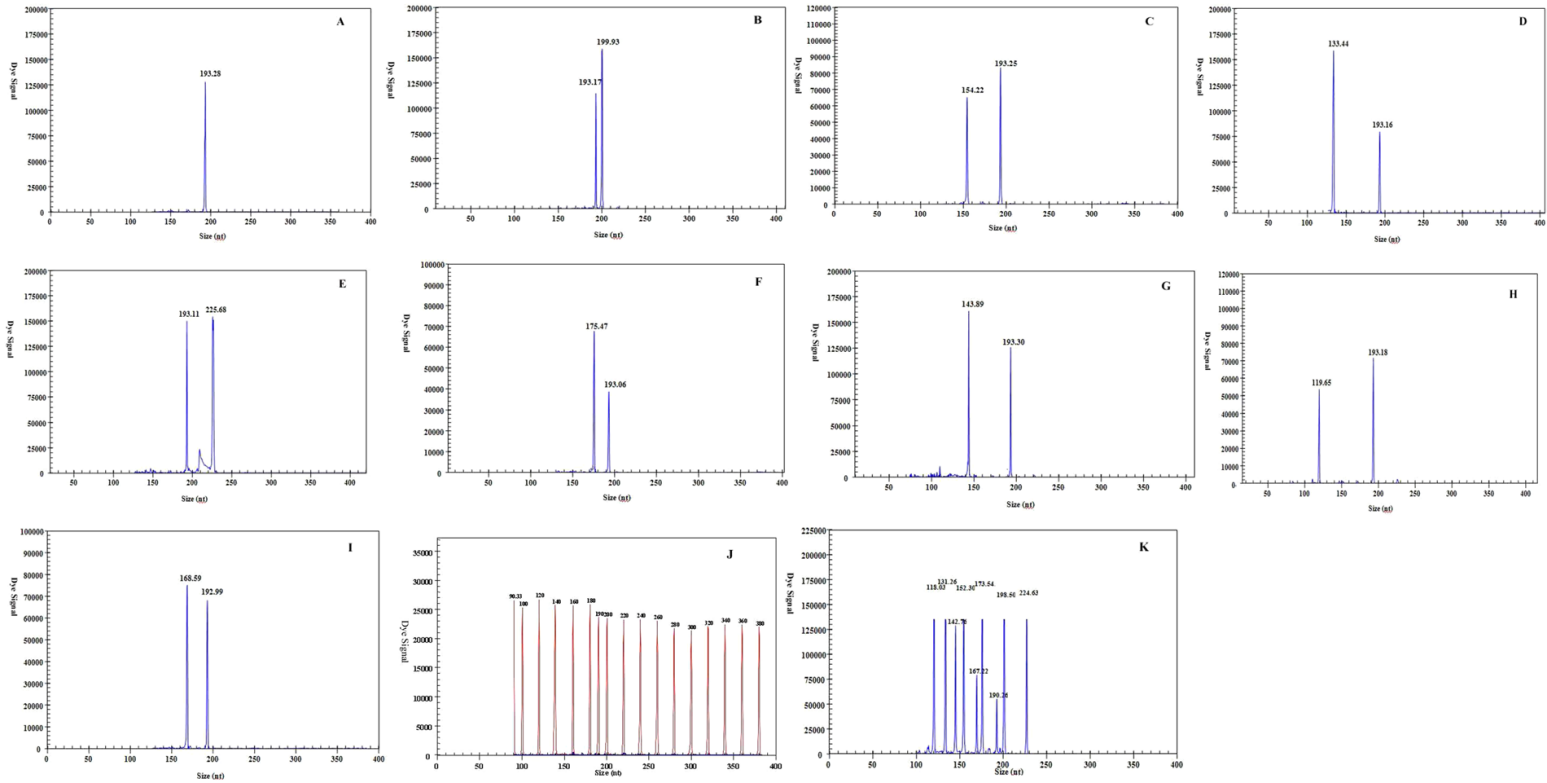
Specificity analyses of GeXP detection of eight avian influenza A viral subtypes with multiplex primers. Cy5-labelled PCR products were separated via GeXP capillary electrophoresis and detected by fluorescence spectrophotometry, given as dye signals in arbitrary units on the y-axis. Each peak was identified by comparing the expected to the actual PCR product size on the x-axis. Panels (A-I and K) show the individual target gene amplification results for M, H1, H2, H3, H5, H6, H7, H9, H10 and all amplified genes simultaneously, respectively. RNase-free water was used as the negative control (J). Red peaks indicate the DNA size standard.

Each pair of gene-specific primers was tested using cDNA samples from eight HA AIV subtypes (H1, H2, H3, H5, H6, H7, H9 and H10) in a mono-PCR-GeXP assay. The target matrix (M) gene was amplified from all AIV subtypes where each specific primer pair could not cross-amplify other HA AIV subtypes but could amplify only the corresponding target gene (Table S1). All amplicon sizes are listed in Table 1. The nine target genes were amplified by the GeXP-multiplex RT-PCR assay, and each primer combination showed specific amplification peaks (Fig. 1). The AIV M gene showed specific amplification peaks for H1N1, H2N3, H3N2, H3N6, H3N8, H4N5, H5N1, H6N1, H6N2, H6N6, H6N8, H7N2, H7N9, H8N4, H9N2, H10N3, H11N3, H12N5, H13N6, H14N5, H15N9 and H16N3.

**Table 1:** GeXP primers designed to detect eight subtypes of the avian influenza A virus.

Primer	Forward primer (5 → 3)a	Reverse primer (5 → 3)a	Size (bp)
M	AGGTGACACTATAGAATAAGCCGAGATCGCGCAGA	GTACGACTCACTATAGGGACGCTCACTGGGCACGGT	192
H1	AGGTGACACTATAGAATACCAGAAYGTGCATCCTATCACT	GTACGACTCACTATAGGGATATCATTCCTGTCCAWCCCCCT	198
H2	AGGTGACACTATAGAATATTCGAGAAAGTRAAGATTYTGCC	GTACGACTCACTATAGGGACCAGACCATGTTCCTGAAGAA	152
H3	AGGTGACACTATAGAATATTGCCATATCATGYTTTTTGCTTTG	GTACGACTCACTATAGGGAAATGCAAATGTTGCACCTAATGTTG	131
H5	AGGTGACACTATAGAATAGGAAAGTGTAAGAAACGGAACGTA	GTACGACTCACTATAGGGA CACATCCATAAAGAYAGACCAGC	223
H6	AGGTGACACTATAGAATA TCTCAAACAAGGCCCCTCTC	GTACGACTCACTATAGGGA TCCCATTTCGGGCATTAGGC	173
H7	AGGTGACACTATAGAATAAGAATACAGATTGACCCAGTSAA	GTACGACTCACTATAGGGA CCCATTGCAATGGCHAGAAG	142
H9	AGGTGACACTATAGAATAACCATTTATTCGACTGTCGCCT	GTACGACTCACTATAGGGACATTGGACATGGCCCAGAA	118
H10	AGGTGACACTATAGAATA AACACGGACACRGCTGA	GTACGACTCACTATAGGGA ATTGTTCTGGTAWGTGGAAC	167
Cy5-Tag-F b	AGGTGACACTATAGAATA		
Tag-R	GTACGACTCACTATAGGGA		

Underlined oligonucleotides are universal sequences.

^a^Degenerate nucleotide abbreviations are as follows: R, A/G; W, A/T; Y, C/T.

^b^The primer is contained in the PCR primer mix.

### GeXP multiplex RT-PCR assay sensitivity

**Figure 2:**
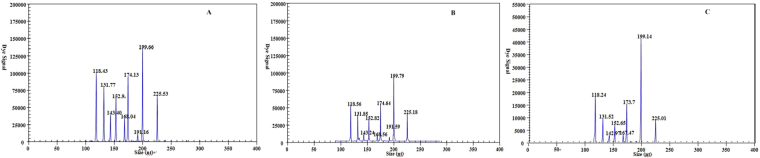
Sensitivity analyses of GeXP detection of nine premixed RNA templates of different concentrations with multiplex primers. Nine different target genes were detected by GeXP at 10^3^ copies/μL (**A**) and 10^2^ copies/μL (**B**). Eight target genes were detected by GeXP at 10^1^ copies/μL (**C**).

For individual ssRNA transcribed *in vitro*, the sensitivity of this method for detecting the AIV type A, AIV-H1, AIV-H2, AIV-H3, AIV-H5, AIV-H6, AIV-H7, AIV-H9 and AIV-H10 templates by the GeXP-multiplex RT-PCR assay was as low as 10^2^ copies/μL (Fig. 2, selected electrophoresis results are shown). Each sample was assayed three times under the same conditions on different days, producing highly similar results.

### Artificial mixture and interference assay

Two specific amplification peaks were observed when two different templates (10^2^ copies of one template and 10^6^ copies of the second template) were evaluated using the GeXP-multiplex RT-PCR assay, and the peak value observed using the single template was similar to that of the mixed template. For example, two specific amplification peaks were observed when different template quantities (from 10^2^ to 10^6^ copies) were tested, and the peak values for these target genes were identical, regardless of whether a single (AIV-H5 or H9) or mixed (AIV-H5 + H9, AIV-H5 + H7 + H9, AIV-H1 + H3 + H6 + H9) template was utilised (electrophoresis results are not shown). These results demonstrated that interference from different templates minimally impacted the detection of a mixed infection.

### Detection of AIVs in clinical samples by the GeXP-multiplex RT-PCR assay

A total of 120 cloacal and oropharyngeal swab samples were randomly collected from poultry and wild birds at various live bird markets (LBMs) from January to May 2017 in Guangxi. All the swab samples were tested for HA AIV serotypes using the optimised GeXP-multiplex RT-PCR assay, and the samples underwent three confirmation RRT-PCR tests, virus isolation and sequencing. The positive and negative results obtained using the various methods are shown in Table 2. Agreement among the GeXP assay, RRT-PCR method and sequencing results is presented in Table 3. The H3, H6 and H9 AIV subtypes were the most common in the 66 positive samples. The GeXP-multiplex RT-PCR assay yielded 100% specificity compared with conventional approaches.

**Table 2:** Detection results for clinical specimens.

Sample No	Typing by RRT-PCR	Conformity	Numbers	GeXP assay								
				M	H1	H2	H3	H5	H6	H7	H9	H10
1–6	H1	+	2	+	+	−	−	−	−	−	−	−
7–21	H3	+	8	+	−	−–	+	−	−	−	−	−
22‒23	H5	+	2	+	−	−	−	+	−	−	−	−
24‒43	H6	+	14	+	−	−	−	−	+	−	−	−
44–48	H7	+	5	+	−	−	−	−	−	+	−	−
49–63	H9	+	14	+	−	−	−	−	−	−	+	−
64	H10	+	1	+	−	−	−	−	−	−	−	+
65–66	H1 + H3	+	2	+	+	−	+	−	−	−	−	−
67–68	H1 + H3 + H6	+	2	+	+	−	+	−	+	−	−	−
69‒71	H3 + H9	+	3	+	−	−	+	−	−	−	+	−
72–76	H6 + H9	+	5	+	−	−	−	−	+	−	+	−
77–78	H5 + H9	+	2	+	−	−	−	+	−	−	+	−
79–100	H2	+	1	+	−	+	−	−	−	−	−	−
101–120	H3 + H6	+	5	+	−	−	−	−	+	−	−	−

All the samples were collected from poultry and wild birds at live bird markets.

**Table 3:** Comparison of the 66 positive clinical samples using the GeXP assay, RRT-PCR methods and sequencing.

Serotype	No. of samples testing positive via:			
	GeXP assay	RRT-PCR	Sequencing	Coincidence rate
H1	2	2	2	100%
H2	1	1	1	100%
H3	8	8	8	100%
H5	2	2	2	100%
H6	14	14	14	100%
H7	5	5	5	100%
H9	14	14	14	100%
H10	1	1	1	100%
Mix	20	20	20	100%
Total	66	66	66	100%

## Discussion

At present, the influenza virus is one of the primary pathogenic microorganisms threatening human health. This virus causes large economic losses and affects social stability. In addition to occasional large influenza outbreaks, certain AIV subtypes cause numerous human deaths each year. In particular, H7N9, which caused an outbreak in China in 2013, has infected more than 1500 humans to date^31^. Influenza viruses contain eight gene segments, and RNA segments of viral strains are prone to gene rearrangement when different influenza strains infect the same cell, resulting in the formation of new strains. Mixed infections with different AIV subtypes are common among birds and appear to play a key role in the natural history of viruses with segmented genomes^32–34^. Multiple AIV subtypes were recently isolated from live poultry markets in southern China^35,36^.

Rapid and accurate identification of AIV HA subtypes is important for understanding AIV circulation in birds and provides useful epidemiological information for selecting appropriate control and elimination strategies. Routine serological tests and primary detection techniques for the influenza virus include RT-PCR, immunofluorescence, virus isolation, and culture methods. However, virus isolation is not always possible due to sample matrix conditions or low viral titres in clinical specimens. These conventional methods for differential diagnosis are complex and time consuming. Moreover, these tests are easily affected by factors such as antibodies. Although new methods for detecting AIV have been developed^37–39^, PCR remains a highly specific and rapid method for accurately detecting pathogens. The GeXP multiplex RT-PCR assay is high-throughput, highly sensitive and specific, can analyse 192 samples in six hours and can simultaneously detect as many as 35 genes in a single PCR reaction. To date, studies have demonstrated that at least eight AIV subtypes can infect humans^15^. These AIVs pose a serious threat to human health and are difficult to differentially and rapidly diagnose using conventional methods. To the best of our knowledge, this study is the first to successfully establish a GeXP multiplex RT-PCR method for rapidly and accurately detecting eight AIV subtypes (H1, H2, H3, H5, H6, H7, H9 and H10) within four hours. This method effectively reduces the time required by the traditional method of identifying each HA subtype individually. The assay avoids cross-reactivity with other HA subtypes of AIV and human seasonal influenza viruses by employing a nucleic-acid-specific validation test and has a detection limit of 10^2^ copies/μL. This assay will greatly accelerates the differential diagnosis of AIV subtypes in infected patients with improved accuracy and thus will be important to develop for use in animal husbandry as well as for human public health.

Because the GeXP multiplex RT-PCR method is highly sensitive, and viral RNA degrades easily, samples and extracted RNA should be stored at −80 °C as quickly as possible to prevent RNA degradation. The high variability of the AIV genome, even within a single HA subtype, causes subtype-specific primer design to be complex; therefore, HA gene sequences were comprehensively selected to consider most circulating isolates. Nonetheless, certain degenerate positions had to be included in the designed primer sequences. More reference strains and clinical samples are needed to validate the reliability of these results due to variations among AIV subtypes. Two distinct advantages of this method are its low cost and short assay time when testing multiple mixed infection samples simultaneously^40,41^. For example, the cost of this GeXP multiplex RT-PCR method for simultaneously detecting eight subtypes of avian influenza A viruses that infect humans is approximately $4 per test, versus $6 per test for each avian influenza A virus using a commercial RRT-PCR kit. The GeXP method cannot be widely used because the system is expensive. By using a one-step RT-PCR kit, the entire reaction can be completed in one tube within 2.5 h, followed by capillary electrophoresis separation. An automated workstation can be employed to reduce the number of steps requiring manual operation to further improve precision, reliability, and speed. Consequently, this method could be immensely helpful in surveillance studies targeting AIV subtypes.

## Methods

### Ethics statement

The present study was approved and conducted in strict accordance with the recommendations in the guide for the care and use of routine sampled animals in LBMs by the Animal Ethics Committee of the Guangxi Veterinary Research Institute, which supervises all LBMs in Guangxi province. Biological samples were gently collected from healthy chickens, ducks, birds and geese using aseptic cotton swabs. The birds were not anaesthetised before sampling, and poultry were observed for 30 min after sampling before being returned to their cages.

### Sample collection and viral DNA/RNA nucleic acid extraction

The pathogens used in this study, which included different AIV subtype reference strains, AIV field isolates and other avian pathogens, are listed in Table S1. All clinical swab samples were collected from the cloacae, larynges and tracheae of healthy chickens, geese and ducks. The sample treatment method was described previously^34^. A viral RNA/DNA Extraction Kit (TaKaRa, Dalian, China) was used to extract genomic RNA/DNA from samples (200 μL of chicken embryo allantoic fluid or liquid cultures) per the manufacturer’s protocol. The extracted RNA was reverse transcribed to synthesise cDNA, and DNA was stored at −80 °C. All samples were manipulated inside a class-II biosafety cabinet in a biosafety level-2 laboratory.

### Primer design and plasmid preparation

The GeXP-multiplex assay consisted of nine gene-specific primer pairs, including one pair of AIV universal primers (AIV M) and eight HA AIV gene segment pairs. Sequence information obtained from the Influenza Sequence Database (http://www.flu.lanl.gov) and the NCBI database were used to design the specific primers. A highly conserved region of the M gene was used to design the AIV universal primers; a specific region of the HA gene segment for all subtypes was used to design the H1, H2, H3, H5, H6, H7, H9 and H10 primers. Premier 6.0, Oligo 7.0 and NCBI Primer BLAST were used for primer analysis and filtration. One universal primer pair was fused at the 5′ end of each gene-specific primer as a universal sequence to generate nine chimeric primer pairs. In addition, we labelled one universal primer pair and one universal primer with Cy5 (Table 1). The primers were purified using high-performance liquid chromatography (HPLC) by Invitrogen (Guangzhou, China).

Plasmids carrying different HA genes from AIV (AIV H1N3 Duck/HK/717/79-d1, AIV H2N3 Duck/HK/77/76, AIV H3N2 A/Chicken/Guangxi/015C10/2009, H5N1 AIV Re-1, AIV H6N8 Duck/HK/531/79, AIV H7N2 AIV Duck/HK/47/76, AIV H9N2 A/chicken/Guangxi/NN1/2011, and AIV H10N3 Duck/HK/876/80) were used for ssRNA synthesis using an RNA production system T7 *in vitro* transcription kit (Promega, Madison, WI, USA). Published methods were used to calculate the ssRNA copy numbers for the target AIV genes (M, H1, H2, H3, H5, H6, H7, H9 and H10)^42^.

### Multiplex PCR reaction conditions for the GeXP analyser

The GeXP PCR reaction system contained a total volume of 25 μL, including 2.5 μL of 10 × PCR Buffer (Sigma, STL, MO, USA), 2.5 μL of MgCl_2_ (25 μM, Sigma), 1.25 μL of universal primers (500 nmol/L), 1.25 μL of JumpStart Taq DNA polymerase (2.5 U/μL, Sigma), 1.25 μL of mixed primers (containing 50–150 nmol/L of nine gene-specific chimeric primer pairs), and 0.5 pg-0.5 ng of template (cDNA or DNA). Next, nuclease-free water was used to bring the final volume of the PCR reaction to 25 μL. The PCR thermocycling procedure was performed at 95 °C for 5 min, followed by 95 °C for 30 s, 55 °C for 30 s and 72 °C for 30 s (10 cycles). The second step was performed at 95 °C for 30 s, 65 °C for 30 s, and 72 °C for 30 s (10 cycles). The third step was performed at 95 °C for 30 s, 55 °C for 30 s, and 72 °C for 30 s (20 cycles). Detailed information regarding the primers is listed in Table 1; all primers were synthesised and purified by Invitrogen (Guangzhou, China).

The GenomeLab GeXP genetic analysis system (Beckman Coulter, Brea, CA, USA) was used to separate and detect the PCR products by capillary electrophoresis following previous protocols^43^. After separating the products, the product peaks were analysed using the GeXP system software. The peak height for each gene is illustrated in an electrophoretogram.

### Single-primer test for specificity

The assay specificity for all target genes was individually assessed using premixed cDNA/DNA in a multiplex PCR assay after optimisation. Other avian pathogens were tested, including all AIV subtypes (except H1, H2, H3, H5, H6, H7, H9 and H10, for which the reference strain HA genes (1–16) were confirmed by sequencing), infectious laryngotracheitis virus (ILTV), Newcastle disease virus (NDV), Abelson leukaemia virus (ALV), infectious bronchitis virus (IBV), *Mycoplasma gallinarum* (MG), avian reovirus (ARV), influenza B viruses, and reticuloendothelial hyperplasia (REV). Nuclease-free water was used as a negative control.

### Evaluation of the sensitivity of the GeXP method and the interference assay

The sensitivity of the multiplex assay was evaluated using a GeXP analyser as previously described^43^. We diluted the same initial concentration for each target gene of eight premixed RNA templates to final concentrations of 10^5^ to 1 copy/μL. Next, PCR products at each dilution were subjected to the multiplex assay using a GeXP system. Finally, specific primer concentrations and amplification systems were optimised based on the best response system. The sensitivity of the GeXP RT-PCR assay was tested three times on three different days in one month using the diluted sample as described previously. Because the simultaneous presence of different template concentrations may affect the amplification efficiency of multiple PCR, several template dilutions (10^2^ to 10^6^ copies/μL) were randomly mixed and tested in the GeXP multiplex RT-PCR assay. The results were also compared with those of the single-template multiplex PCR assay.

### Applications for detecting clinical specimens

We randomly collected 120 clinical specimens from different poultry and wild bird species in LBMs, and all clinical specimens were subjected to virus isolation, GeXP-multiplex RT-PCR and RRT-PCR simultaneously. Using previously reported primers, HA genes from positive specimens in the methods described above were sequenced by BGI (Shenzhen, China) to confirm RT-PCR compliance^44^.

## Electronic supplementary material

Dataset 1
