# Supplementary material for: Simultaneous detection of eight avian influenza A virus subtypes by multiplex reverse transcription-PCR using a GeXP analyser
Source: Sci Rep. 2018 Apr 18;8:6183. doi: 10.1038/s41598-018-24620-8 (PMC5906657; doi:10.1038/s41598-018-24620-8)
Supplement: Supplementary file 1 — Dataset 1 [file 41598_2018_24620_MOESM1_ESM.doc]

**Simultaneous detection of eight avian influenza A virus subtypes by multiplex reverse transcription-PCR using a GeXP analyser**

Meng Li, Zhixun Xie*, Zhiqin Xie, Jiabo Liu, Liji Xie, Xianwen Deng, Sisi Luo, Qing Fan, Li Huang, Jiaoling Huang, Yanfang Zhang, Tingting Zeng, Sheng Wang

***Guangxi Key Laboratory of Veterinary Biotechnology,*** ***Guangxi Veterinary Research Institute,*** ***51 You Ai North Road,*** ***Nanning, Guangxi*** ***530001,*** ***China.***

*[Corresponding](mailto:Corresponding) [author](app:ds:author): xiezhixun@126.com.

| Pathogens/field samples | Numbers of sample | Source | Results | | | | | | | | |
| --- | --- | --- | --- | --- | --- | --- | --- | --- | --- | --- | --- |
|  |  |  | M | H1 | H2 | H3 | H5 | H6 | H7 | H9 | H10 |
| Reference samples |  |  |  |  |  |  |  |  |  |  |  |
| AIV H1N3 Duck/HK/717/79-d1 | 1 | HKU | + | + | - | - | - | - | - | - | - |
| AIV H1N1 Human/NJ/8/76 | 1 | HKU | + | + | - | - | - | - | - | - | - |
| AIV H2N3 Duck/HK/77/76 | 1 | HKU | + | - | + | - | - | - | - | - | - |
| AIV H3N6 AIV Duck/HK/526/79/2B | 1 | HKU | + | - | - | + | - | - | - | - | - |
| AIV H3N2 A/Chicken/Guangxi/015C10/2009 | 1 | GVRI | + | - | - | + | - | - | - | - | - |
| AIV H3N2 A/Duck/Guangxi/015D2/2009 | 1 | GVRI | + | - | - | + | - | - | - | - | - |
| AIV H3N6 A/pigeon/Guangxi/020P/2009 | 1 | GVRI | + | - | - | + | - | - | - | - | - |
| AIV H3N6 A/Duck/Guangxi/175D12/2014 | 1 | GVRI | + | - | - | + | - | - | - | - | - |
| Inactivated H5N1 AIV Re-1 | 1 | HVRI | + | - | - | - | + | - | - | - | - |
| cDNA of H5N3 AIV Duck/HK/313/78 | 1 | CU | + | - | - | - | + | - | - | - | - |
| cDNA of AIV H5N2/chicken/QT35/87 | 1 | CU | + | - | - | - | + | - | - | - | - |
| cDNA of AIV H5N5/chicken/QT35/98 | 1 | CU | + | - | - | - | + | - | - | - | - |
| cDNA of AIV H5N7 A/waterfowl/GA/269452-56/03 | 1 | CU | + | - | - | - | + | - | - | - | - |
| cDNA of AIV AIV H5N9/chicken/QT35/98 | 1 | CU | + | - | - | - | + | - | - | - | - |
| Inactivated H5N1 AIV Re-4 | 1 | HVRI | + | - | - | - | + | - | - | - | - |
| Inactivated H5N1 AIV Re-5 | 1 | HVRI | + | - | - | - | + | - | - | - | - |
| Inactivated H5N1 AIV Re-6 | 1 | HVRI | + | - | - | - | + | - | - | - | - |
| Inactivated H5N1 AIV Re-7 | 1 | HVRI | + | - | - | - | + | - | - | - | - |
| Inactivated H5N1 AIV Re-8 | 1 | HVRI | + | - | - | - | + | - | - | - | - |
| AIV H6N8 Duck/HK/531/79 | 1 | HKU | + | - | - | - | - | + | - | - | - |
| AIV H6N1 A/Duck/Guangxi/GXd-5/2010 | 1 | GVRI | + | - | - | - | - | + | - | - | - |
| AIV H6N1 A/duck/Guangxi/105/2011 | 1 | GVRI | + | - | - | - | - | + | - | - | - |
| AIV H6N2 A/goose/Guangxi/105/2011 | 1 | GVRI | + | - | - | - | - | + | - | - | - |
| AIV H6N2 A/goose/Guangxi/115/2012 | 1 | GVRI | + | - | - | - | - | + | - | - | - |
| AIV H6N2 A/duck/Guangxi/116/2012 | 1 | GVRI | + | - | - | - | - | + | - | - | - |
| AIV H6N2 A/chicken/Guangxi/121/2013 | 1 | GVRI | + | - | - | - | - | + | - | - | - |
| AIV H6N2 A/duck/Guangxi/121/2012 | 1 | GVRI | + | - | - | - | - | + | - | - | - |
| AIV H6N6 A/duck/Guangxi/058/2010 | 1 | GVRI | + | - | - | - | - | + | - | - | - |
| AIV H6N6 A/chicken/Guangxi/129/2013 | 1 | GVRI | + | - | - | - | - | + | - | - | - |
| AIV H6N6 A/duck/Guangxi/131/2013 | 1 | GVRI | + | - | - | - | - | + | - | - | - |
| AIV H6N6 A/pigeon/Guangxi/161/2014 | 1 | GVRI | + | - | - | - | - | + | - | - | - |
| AIV H6N6 A/Duck/Guangxi/GXd-7 /2011 | 1 | GVRI | + | - | - | - | - | + | - | - | - |
| AIV H6N8 A/Duck/Guaiigxi/GXd-6/2010 | 1 | GVRI | + | - | - | - | - | + | - | - | - |
| AIV H6N8 A/duck/Guangxi/113/2012 | 1 | GVRI | + | - | - | - | - | + | - | - | - |
| cDNA of AIV H7N2 AIV Duck/HK/47/76 | 1 | HKU | + | - | - | - | - | - | + | - | - |
| cDNA of AIV H7N2/chicken PA/3979/97 | 1 | PU | + | - | - | - | - | - | + | - | - |
| cDNA of AIV H7N9 A/chicken/Rizhao/875/2013 | 1 | HKU | + | - | - | - | - | - | + | - | - |
| cDNA of AIV H7N9 A/chicken/Zhejiang/SD007/2013 | 1 | HVRI | + | - | - | - | - | - | + | - | - |
| cDNA of AIV H7N9 A/Chicken/Guangxi/YL2/2017 | 1 | GVRI | + | - | - | - | - | - | + | - | - |
| cDNA of AIV H7N9 A/Chicken/Guangxi/NN1/2017 | 1 | GVRI | + | - | - | - | - | - | + | - | - |
| cDNA of AIV H7N9 A/Chicken/Guangxi/YL1/2017 | 1 | GVRI | + | - | - | - | - | - | + | - | - |
| AIV H9N2 A/turtledove/Guangxi/49B6/2013 | 1 | GVRI | + | - | - | - | - | - | - | + | - |
| AIV H9N2 A/chicken/Guangxi/NN2/2011 | 1 | GVRI | + | - | - | - | - | - | - | + | - |
| AIV H9N2 A/chicken/Guangxi/NN1/2011 | 1 | GVRI | + | - | - | - | - | - | - | + | - |
| AIV H9N2 A/chicken/Guangxi/111C8/2012 | 1 | GVRI | + | - | - | - | - | - | - | + | - |
| AIV H9N2 A/chicken/Guangxi/116C4/2012 | 1 | GVRI | + | - | - | - | - | - | - | + | - |
| AIV H9N2 A/pheasant/Guangxi/49B2/2013 | 1 | GVRI | + | - | - | - | - | - | - | + | - |
| AIV H9N2 A/sparrow/Guangxi/35B15/2013 | 1 | GVRI | + | - | - | - | - | - | - | + | - |
| AIV H9N2 A/dove/Guangxi/31B6/2013 | 1 | GVRI | + | - | - | - | - | - | - | + | - |
| AIV H9N2 A/chicken/Guangxi/141C10/2013 | 1 | GVRI | + | - | - | - | - | - | - | + | - |
| AIV H9N2 A/chicken/Guangxi/CX/2013 | 1 | GVRI | + | - | - | - | - | - | - | + | - |
| AIV H9N2 A/chicken/Guangxi/LF2/2014 | 1 | GVRI | + | - | - | - | - | - | - | + | - |
| AIV H9N2 A/quail/Guangxi/210Q33/2015 | 1 | GVRI | + | - | - | - | - | - | - | + | - |
| AIV H9N2 A/partridge/Guangxi/116B13/2015 | 1 | GVRI | + | - | - | - | - | - | - | + | - |
| AIV H9N2 A/sparrow/Guangxi/130B2/2015 | 1 | GVRI | + | - | - | - | - | - | - | + | - |
| AIV H9N2 A/pheasant/Guangxi/156B7/2016 | 1 | GVRI | + | - | - | - | - | - | - | + | - |
| AIV H9N2 A/sparrow/Guangxi/160B8/2016 | 1 | GVRI | + | - | - | - | - | - | - | + | - |
| AIV H10N3 Duck/HK/876/80 | 1 | HKU | + | - | - | - | - | - | - | - | + |
| AIV H4N5 Duck/HK/668/79 | 1 | HKU | + | - | - | - | - | - | - | - | - |
| AIV H8N4 AIV Turkey/ont/6118/68 | 1 | HKU | + | - | - | - | - | - | - | - | - |
| AIV H11N3 Duck/HK/661/79 | 1 | HKU | + | - | - | - | - | - | - | - | - |
| AIV H12N5 Duck/HK/862/80 | 1 | HKU | + | - | - | - | - | - | - | - | - |
| AIV H13N5 AIV Gull/MD/704/77 | 1 | HKU | + | - | - | - | - | - | - | - | - |
| AIV H13N6 A/Gull/Maryland/704/1977 | 1 | PU | + | - | - | - | - | - | - | - | - |
| AIV H14N5 A/Mallard duck/Astrakhan/263/1982 | 1 | PU | + | - | - | - | - | - | - | - | - |
| AIV H15N9  A/wedge-tailed shearwater/Western Australia/2576/1979 | 1 | PU | + | - | - | - | - | - | - | - | - |
| AIV H16N3 A/shorebird/Delaware/168/06 | 1 | PU | + | - | - | - | - | - | - | - | - |
| IAV H1N1 A/ Guangxi/1415/15 | 1 | GCDC | - | - | - | - | - | - | - | - | - |
| IAV H3N2 A/ Guangxi/1632/15 | 1 | GCDC | - | - | - | - | - | - | - | - | - |
| B/Guangxi/1470/15 | 1 | GCDC | - | - | - | - | - | - | - | - | - |
| Other pathogens |  |  |  |  |  |  |  |  |  |  |  |
| NDV Lasota | 1 | CIVDC | - | - | - | - | - | - | - | - | - |
| IBV Massachussetts 41 | 1 | CIVDC | - | - | - | - | - | - | - | - | - |
| ILTV(AV1231) | 1 | GVRI | - | - | - | - | - | - | - | - | - |
| MG S6 | 1 | GVRI | - | - | - | - | - | - | - | - | - |
| MS K1415 | 1 | GVRI | - | - | - | - | - | - | - | - | - |
| HPG AV269 | 1 | GVRI | - | - | - | - | - | - | - | - | - |
| Avian reovirus(S1133) | 1 | GVRI | - | - | - | - | - | - | - | - | - |

**Table S1. Sources of pathogens used and GeXP assay results.**

HVRI = Harbin Veterinary Research Institute, China.

HKU = The University of HongKong, China.

GVRI = Guangxi Veterinary Research Institute, China.

CIVDC = China Institute of Veterinary Drugs Control, China.

PU = University of Pennsylvania, USA.

GCDC=  Guangxi Center for Disease Control.

CU= University of Connecticut, USA.
